# Modifications of the Effect of Juvenile Idiopathic Arthritis (JIA) on Anxiety and Depression in Children and Adolescents: A Pseudo-longitudinal Study of 192,019 Children in the United States

**DOI:** 10.2174/0117450179372640250324051404

**Published:** 2025-04-11

**Authors:** Kira Gor, Yu-Sheng Lee, Matthew Evan Sprong, Heaven Hollender, Junu Shrestha, Xueli Huang

**Affiliations:** 1 School of Integrated Sciences, Sustainability, and Public Health, College of Health, Science, and Technology, University of Illinois at Springfield, Springfield, United States; 2 School of Public Management and Policy, College of Public Affairs and Education, University of Illinois at Springfield, Springfield, United States; £ Present Address: Department of Addictions Studies and Behavioral Health, College of Health and Human Services, Governors State University, University Park, IL, United States; 3 School of Health and Human Sciences, Indiana University Indianapolis, Indianapolis, United States; 4 Department of Computer Science, College of Health, Science, and Technology, University of Illinois at Springfield, Springfield, United States

**Keywords:** Juvenile idiopathic arthritis, Depression, Anxiety, Mental health, Insurance, Children with disabilities, Effect modification, Augmented backward elimination, NSCH

## Abstract

**Introduction:**

Juvenile Idiopathic Arthritis (JIA), among children and adolescents, is a heterogeneous condition and is a prevalent chronic rheumatological disease. Non-medical (*e.g.*, self-efficacy, social support, parental distress, and coping with pain), medical factors (*e.g.*, permanent damage to joints), and psychological factors (*e.g.*, depression and anxiety) can significantly impact the quality of life for individuals with JIA.

**Methods:**

This study aimed to investigate the effect modifiers of the associations of anxiety and depression in children with JIA. The National Survey of Children’s Health database (2016-2021) was used for the current study. A total of 192,019 children were included in the analyses. An augmented backward elimination model selection method was used to identify predictors for depression and anxiety.

**Results:**

The period prevalence of JIA was 2.723 per 1,000. Sex was an effect modifier. Among boys, those who had JIA were 2.96 times (p<0.0001) more likely to have depression compared to non-JIA boys. On the other hand, the effects of JIA on anxiety were different across the insurance types. Among children with public insurance, children with JIA were 6.28 times (p <0.0001) more likely to have anxiety than those without JIA. Among children with JIA, those with public insurance were 5.23 times (p = 0.0005) more likely to have anxiety than those with private insurance.

**Discussion:**

This population-based study found that typical sex differences in depression were not observed in the JIA group and that children with JIA had higher rates of anxiety, particularly those with public insurance. These findings highlight the need for integrated care that addresses both physical and mental health.

Collaborative models involving rheumatologists and mental health professionals may aid in early intervention. Limitations include the study’s cross-sectional design, which did not establish a causal association, and a lack of analysis by the JIA subtype, which could have varying impacts on mental health outcomes.

**Conclusion:**

The findings highlight the importance of conducting comprehensive mental health assessments and developing personalized interventions tailored to the needs of JIA patients. The observed sex differences and the impact of insurance type on anxiety further emphasize the necessity of individualized care approaches.

## 1 INTRODUCTION

Juvenile idiopathic arthritis (JIA) is a common chronic rheumatological disease in children and adolescents, characterized by persistent joint inflammation lasting at least six weeks with onset before age 16 [1-5].

### 1.1 JIA Epidemiology

Reported prevalence estimates range from 12.8 to 45 per 100,000, and incidence rates from 7.8 to 8.3 per 100,000 person-years [6-11], although these may be underestimates [4]. JIA subtypes include oligoarticular, polyarticular (RF-positive or RF-negative), enthesitis-related, psoriatic, systemic, and undifferentiated arthritis [5, 11]. JIA can substantially affect both physical and psychological health, impacting the quality of life and increasing healthcare costs [12-14]. A systematic review observed that annual costs for JIA can vary widely from $310 up to $44,832 per patient, largely depending on factors such as the country of treatment, disease activity, JIA subtype, and use of biological therapies [12]. Although “juvenile” typically implies childhood onset, a significant proportion of children with JIA experience ongoing inflammation into adolescence and adulthood, *i.e.*, more than a third of children with JIA continue to experience active inflammation throughout their adult years [15-17].

JIA rarely occurs in babies younger than six months [18-20]. Peak onset ages for JIA often occur between 2-5 years and again between 6-14 years [4, 19, 21, 22]. JIA has also sex differences, with girls diagnosed more frequently than boys, especially with the oligoarticular and polyarticular RF-negative subtypes [4, 13, 23, 24]. The oligoarticular subtype is the most common JIA subtype in developed countries, typically affecting girls under six years old [24]. On the other hand, the polyarticular JIA-RF negative showed a bimodal trend in girls, *i.e.*, peaks at 2 - 4 years and 6 - 12 years, which is consistent in general for typical JIA diagnosing onset age [4].

### 1.2 Sex, Medical Insurance, and Psychological Comorbidities in JIA

Children with JIA often face multiple psychosocial challenges. Among these, depression and anxiety are two of the most frequently observed mental health concerns [13, 25-27]. Their prevalence ranges widely, from 7% to 36% for depression and 7% to 64% for anxiety [13]. These mental health symptoms can arise from chronic pain, reduced mobility, social isolation, and uncertainty about long-term outcomes [14, 18, 28, 29].

The sex differences in mental health are well established, with adolescent girls typically demonstrating higher rates of depression than boys [30-32]. However, studies focusing specifically on anxiety and depression among children with JIA remain limited, frequently involving small samples and a few minority participants [13, 25, 33-36].

Socioeconomic status (SES), often reflected in medical insurance status, is another important factor. Families with public insurance (frequently associated with lower SES) may face more barriers to comprehensive healthcare, potentially leading to delayed or limited treatment. By contrast, children with private insurance usually have greater access to specialized care, potentially mitigating the psychological burden [37].

### 1.3 Study Objectives

In this context, the primary objective of our study was to examine the association between JIA and two key mental health outcomes, depression and anxiety, in a large, nationally representative sample. Specifically, we sought to 1) assess the prevalence of JIA, depression, and anxiety in children in the United States (U.S.), 2) identify significant predictors (demographic, clinical, psychosocial) for depression and anxiety in children with and without JIA), and 3) investigate effect modification by sex in the JIA depression association and by insurance type in the JIA anxiety association. By illuminating these relationships, we aim to guide more tailored interventions, including mental health screening and support strategies, for children living with JIA.

## 2 METHODS

### 2.1 Source of Data

The National Survey of Children’s Health (NSCH) database (January 1, 2016 – December 31, 2021) was used for the current study. The NSCH database is funded by the Health Resources and Services Administration and Child Health Bureau to collect physical and mental health, access to quality health care, and the child’s family, neighborhood, school, and social context information [37, 38] of children ages 0 to 17 surveyed in all 50 states plus the District of Columbia. Surveys are conducted *via* mail and web-based surveys by the U.S. Census Bureau between 2016 and 2021, including the prior version of the NSCH and a second survey that includes questions related to children with special needs: “National Survey of Children with Special Health Care Needs” (NS-CSHCN). The NSCH compared response rates across various demographic and socioeconomic subgroups to highlight disparities. The analysis examined the effectiveness of weighting adjustments to reduce nonresponse bias. The weighting process for interviewed children started with a base weight for each sampled household, followed by a nonresponse adjustment for the screener. Eligible children were then adjusted using a Child-Level Screener Factor and a within-household subsampling factor. A nonresponse adjustment for topical issues was applied, followed by a ranking adjustment to demographic controls and trimming of extreme weights if necessary. Although findings indicated some differences between respondents and nonrespondents, the weighting adjustments were generally effective in minimizing the nonresponse bias and enhancing the survey's representativeness [38-41]. Additional information about the sampling and administration process, survey methodology, nonresponse bias analysis, and other pertinent information can be found on the survey’s website [38].

### 2.2 Study Population

The 2016 NSCH was conducted from June 2016 through February 2017; the 2017 NSCH was conducted between August 2017 and February 2018; the 2018 NSCH was conducted between June 2018 and January 2019; the 2019 NSCH was conducted between June 2019 and January 2020; the 2020 NSCH was conducted between June 2020 and January 2021; and the 2021 NSCH was conducted between July 2021 and January 2022. Additional information on the sampling and administration process, survey methodology, nonresponse bias analysis, and other pertinent information can be found on the survey’s website [37]. Since 2016, NSCH data files can be combined to increase the analytic sample size and investigate the time-series trend with multiple years of non-overlapping sampling data [38]. JIA is defined as arthritis of unknown etiology that begins before age 16 [1-5]. Nevertheless, some studies and healthcare systems have expanded this definition to encompass individuals up to 18 years old to be consistent with broader pediatric categories [39]. Although symptoms can appear as early as infancy, identifying JIA in children under one year is relatively rare; nonetheless, there have been cases of diagnosis prior to their first birthday [40]. Moreover, the NSCH collected data on JIA for children under 17 years of age. Therefore, we included all children aged 0 to 17 as the study population. After approval by the institutional review board of the primary author’s university (approval #24-053), 223,195 children between 0 and 17 from the NSCH database were examined. Because the questions on anxiety problems and depression in NSCH only included children between three and seven years, 31,176 children under three years old were excluded from the current study, resulting in 192,019 remaining in the analyses. This included 550 JIA cases, and the distribution of these children’s demographic characteristics is presented in Table **1**.

### 2.3 Predictors and Outcomes

The primary outcomes of this study are depression and anxiety. The questions that NSCH asked children between ages 3 and 17 about depression and anxiety were, “Does this child have this current or lifelong health condition?” The NSCH identified JIA by asking, *“Has a doctor or other health care provider EVER told you that this child has arthritis?”* and “*How severe are this child’s conditions if the child has current or lifelong conditions?*” Those responses for currently having the condition and the severity was mild, moderate, or severe were considered to have arthritis [11].

The predictors included the baseline information on children’s age, sex, race/ethnicity, maternal age at delivery, premature birth, low birth weight, months of breastfeeding, body mass index (BMI), sleeping less than recommended age-appropriate hours, allergic to food, drug, and/or insect, has asthma, history of a brain injury, had behavior or conduct problem, had development delay, intellectual disability, speech disability, learning disability, frequency of physical activity, had difficulties in making or keeping friends, insurance type, father’s physical and mental health status, mother’s physical and mental health status, parent or guardian divorced or separated, child’s parent or guardian died, child’s parent or guardian served time in jail or prison, child witnessed domestic violence, child was victim or witness of neighborhood violence, child lived with anyone who was mentally ill, suicidal, or severely depressed, child lived with anyone who had a problem with alcohol or drug, and child was treated or judged unfairly because of their race or ethnic group.

### 2.4 Statistical Analysis Methods

We summarized continuous variables with the mean (standard deviation) and presented categorical variables as frequency (percent). We evaluated differences between continuous variables with a t-test or a Mann-Whitney U test as the normality assumption was unmet. The associations between categorical variables were estimated with the Chi-square or Fisher’s Exact test. A false discovery rate (FDR) of 0.05 was applied to adjust for the inflation of significance levels due to multiple comparisons. The period prevalence was calculated using the SAS SURVEY procedure to represent the population of noninstitutionalized children nationally and in each state [38, 42].

We used an Augmented Backward Elimination (ABE) model selection method to identify predictors for depression and anxiety using the R *ABE* package [43]. The ABE method accommodates various model types, such as logistic, Cox, and linear regressions. We used logistic regressions for this current study. The ABE selection method uses a Change-in-Estimate criterion (τ), indicating that a variable should be included in the model if its addition significantly alters the estimate of other variables [43-45]. The ABE procedure started with a full model that included all potential predictors and evaluated the *p-value*s of all effects. Variables with a mild significant level of 0.2 were ranked and sequentially reintroduced individually to test for confounding effects. This assessment involved examining how the inclusion of the previously excluded variable affected the estimates of other variables within the model [43, 46]. Variables with a significant Change-in-Estimate criterion (τ) greater than 0.05 are excluded from the model [43]. The full models for depression and anxiety included the significant variables in Table **1**. The ABE model selection method allows the user to retain the critical variables in the model, preventing removals. In this current study, we retained JIA in the models during the selections. After we selected the parsimonious models for depression and anxiety, we further tested the interaction term.

We performed a post hoc power analysis, which showed an average achieved power of 74.1%, confirming that our sample size was adequate for detecting significant effects. This pseudo-longitudinal (repeated cross-sectional) study was conducted in accordance with the Strengthening the Reporting of Observational Studies in Epidemiology (STROBE) guidelines to ensure methodological rigor and transparency in reporting. All analyses were performed in SAS package version 9.4 (SAS Institute Inc, NC) or R package version 4.2.2 (R Core Team 2023. R: A Language and Environment for Statistical Computing. R Foundation for Statistical Computing, Vienna. https://www.R-project.org/).

## 3 RESULTS

### 3.1 Study Sample Characteristics

There were 192,019 children collected in the NSCH database from 2016 to 2021. We found that the period prevalence of JIA was 2.723 per 1,000. Table **1** demonstrates the demographics and health status of the two groups (children with *vs*. without JIA). In total, 550 children diagnosed with JIA [213 (38.7%) boys; 337 (61.3%) girls] were reported between 2016 and 2021. The mean age of children with JIA was 13.40 (SD=3.66) years. The median (IQR) age of children with JIA was 14 (11, 16). Mothers’ age at delivery was significantly younger in children with JIA compared to children without JIA (29.1 *vs*. 30.1, p=0.0004). Children with JIA had a higher percentage of reported depression (21.5% *vs*. 4.5%, p<0.0001) and anxiety (36.6% *vs*. 10.5%, p<0.0001) than children without JIA. The period prevalence of depression among children with JIA compared to those without JIA was 231.12 *vs*. 35.92 per 1,000, while the prevalence of anxiety was 384.32 *vs*. 81.88 per 1,000. Children with JIA were also more likely to have allergies to food, drugs, or insects (48.8% *vs*. 23.7%, p<0.0001), to have asthma (25.7% *vs*. 8.4%, p<0.0001), developmental delay (16.8% *vs*. 5.5%), learning disability (22.9% *vs*. 7.3%, p<0.0001), being more challenging to make or keep friends (15.5% *vs*. 4.0%, p<0.0001). The frequency of no physical activity (0 days per week) was found to be higher in children with JIA compared to non-JIA ones (18.6% *vs*. 7.5%, p<0.0001). The insurance distribution was different between JIA and non-JIA children, *i.e.*, 71.3% of non-JIA children had private insurance, while 53.3% of children with JIA had private insurance. On the contrary, public insurance had more children with JIA than without JIA ones (33.5% *vs*. 20.3%).

### 3.2 Risk Factors for Depression and Effect Modification

Table **2** shows the risk factors for depression. In the depression model, the ABE selected risk factors included children’s age, sex, race, maternal age at delivery, premature birth, months of breastfeeding, overweight or obese, sleeping less than recommended age-appropriate hours, allergic to food, drug, or insect, asthma, behavior or conduct problem, developmental delay, intellectual disability, speech disability, learning disability, physical activity, difficulties in making or keeping friends, insurance type, parents divorced or separated, victim or witness of neighborhood violence, lived with anyone who was mentally ill, suicidal, or severely depressed. Brain injury and JIA were not predictors for depression (Odds Ratio (OR) = 1.26, p = 0.2356; OR = 1.22, p = 0.4310).

**Fig. (1):**
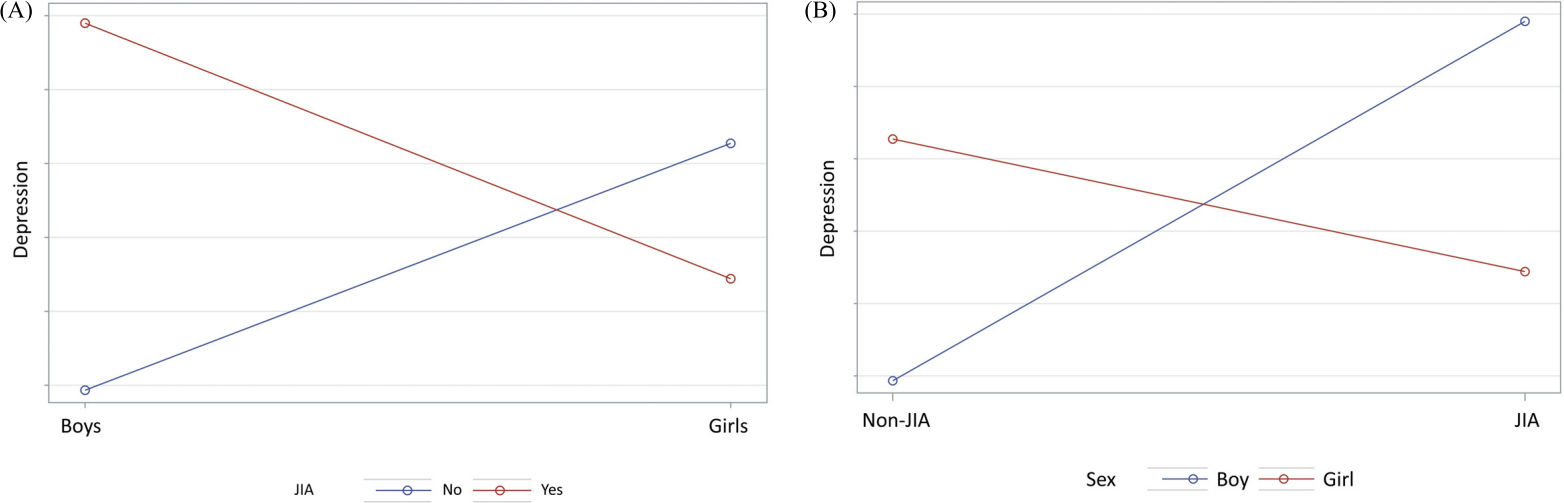
Interaction of JIA and sex. Sex is an effect modifier. In (**A**), among boys, those who had JIA were more likely to have depression compared to non-JIA boys. There was no difference in having depression between JIA and non-JIA among girls. In (**B**), among children with JIA, there was no difference between boys and girls in having depression. However, among children without JIA, boys were less likely to have depression than girls.

However, we found an interaction term between JIA and sex (p<0.0001), *i.e.*, sex is an effect modifier in the association of JIA and depression. Thus, JIA should be kept in the model. Specifically, among boys, those who had JIA were 2.96 times (95% C.I. = 1.43-6.13) more likely to have depression compared to non-JIA boys. There was no difference in having depression between JIA and non-JIA among girls (p = 0.2529) (Fig. **1A**). Among children with JIA, there was no difference between boys and girls in having depression (Fig. **1B**). Among children without JIA, boys were less likely to have depression than girls (OR = 0.51, 95% C.I. = 0.47-0.57, p<0.0001).

### 3.3 Risk factors for anxiety and Effect modification

JIA was found to be a risk factor for children's anxiety (Table **3**). That is, children with JIA were 1.66 times (95% C.I = 1.13-2.44, p = 0.0092) more likely to have anxiety than children without JIA. Other risk factors for anxiety include premature birth, months of breastfeeding, sleeping less than recommended age-appropriate hours, allergic to food, drugs, or insects, asthma, brain injury, having behavior or conduct problems, having a learning disability, with difficulties in making or keeping friends, victim or witness of neighborhood violence, lived with anyone who was mentally ill, suicidal, or severely depressed, and treated or judged unfairly because of their race or ethnic group. Sex was a risk factor for JIA. Boys were less likely to have anxiety (OR = 0.58, p <0.0001). However, we did not find an interaction between sex and anxiety.

**Fig. (2):**
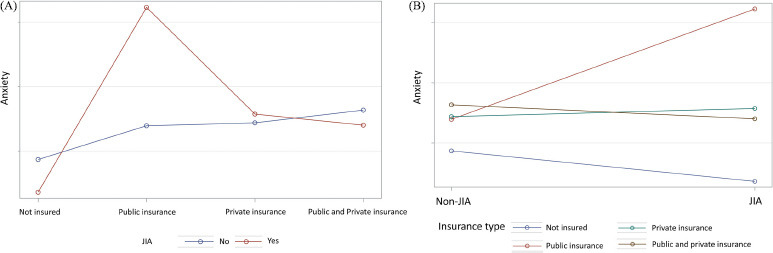
Interaction of JIA and insurance. The effects of JIA on anxiety were different across the insurance types. In (**A**), among children with JIA, those with public insurance were more likely to have anxiety than those with private insurance. In (**B**), among children with public insurance, children with JIA were more likely to have anxiety than children without JIA.

On the other hand, we found that the effects of JIA on anxiety were different across the insurance types. Specifically, among children with public insurance, children with JIA were 6.28 times (95% C.I. = 2.83-13.90, p <0.0001) more likely to have anxiety than children without JIA (Table **3** and Fig. **2A**). Among children with JIA, those with public insurance were 5.23 times (95% C.I. = 2.07-13.25, p = 0.0005) more likely to have anxiety than those with private insurance (Table **3** and Fig. **2B**).

## 4 DISCUSSION

This large, population-based, cross-sectional study examined whether JIA is associated with elevated rates of depression or anxiety. Further, it assessed the modification effects of sex (for depression) and insurance types (for anxiety). Of the 192,019 children in the analysis, 550 JIA cases (2.723 per 1,000) were reported, with 213 (38.7%) boys and 337 (61.3%) girls. The period prevalences of depression and anxiety among children with JIA were 231.12 per 1,000 and 384.32 per 1,000, respectively. These prevalences were higher than that in children without JIA. We also found that among boys, JIA was strongly associated with a higher prevalence of depression (OR = 2.96). Notably, among children without JIA, boys had a lower risk of depression than girls (OR = 0.51), an expected result. However, this “protective” effect of being male was absent in JIA, suggesting that JIA may eliminate typical sex disparities in depression. While research consistently reports higher depression rates in adolescent girls [23-25], fewer large-scale data exist on sex-specific psychological outcomes in pediatric arthritis. Our results underscore the need for mental health screening in all children with JIA, especially boys, who might otherwise be overlooked. The uniqueness of our findings highlights that we are among the first to emphasize this aspect, stressing the necessity for additional research to verify and delve into these sex differences more thoroughly.

Children with JIA were more likely to have anxiety (OR =1.66) than those without JIA overall, but the effect was significantly heightened among those with public insurance (OR = 6.28). Insurance type can be a proxy for socioeconomic status and healthcare accessibility [26]. Children from lower socioeconomic backgrounds may have limited access to specialists or consistent treatment, exacerbating worry and uncertainty. Conversely, those uninsured may have lower reported diagnosis rates of anxiety due to reduced healthcare contact. Future interventions are suggested to incorporate socioeconomic determinants, ensuring that vulnerable children (*e.g.*, on public insurance) receive comprehensive mental health support [37, 47, 48].

### 4.1 Interventions for Children with JIA

Given these findings, interventions should address both the clinical management of JIA and the psychological challenges these children face. Collaborative care models, where pediatric rheumatologists, mental health professionals, and social workers coordinate, may help detect and treat anxiety or depression early [49]. One promising strategy is Cognitive Behavioral Therapy (CBT), which supports children with chronic pain by helping them develop coping skills and manage negative thought patterns and stress [50]. In addition, telemedicine and app-based interventions offer accessible mental health support and symptom-tracking tools, particularly for children with public insurance who face healthcare barriers [51]. Another viable approach is Mindfulness-Based Stress Reduction (MBSR), which can enhance coping and emotional regulation; this technique has shown success in pediatric populations managing chronic pain [52, 53]. Meanwhile, integrated psychoeducation programs guide parents and children in recognizing symptom changes, adhering to medication plans, and employing stress-reduction strategies, all of which can positively influence health outcomes [54]. Addressing low SES barriers entails forming partnerships with community organizations and broadening insurance coverage for mental health services, thus mitigating financial and logistical obstacles to care [55]. By incorporating these strategies, healthcare systems can significantly reduce the mental health burden among children with JIA and minimize downstream complications related to both disease progression and untreated psychiatric conditions.

### 4.2 Strengths and Limitations

This study’s major strength lies in its large, nationally representative sample, enhancing statistical power and generalizability. By utilizing the NSCH dataset from 2016 to 2021, we were able to obtain a representative study sample size that significantly increased the statistical power of our research. This allowed us to delve deeper into the multifaceted impact of JIA on affected children and their families, providing a comprehensive analysis of how JIA influences both physical and mental health outcomes. The large sample size also enabled us to explore various demographic, clinical, and psychosocial factors in greater detail, shedding light on the complex interplay between these variables and mental health issues in children with JIA. Our findings underscore the importance of addressing JIA patients' physical and psychological needs to improve their overall well-being and quality of life. Future research should continue to build on these insights, focusing on longitudinal studies to better understand JIA's causal relationships and long-term effects on mental health.

Despite the strengths of this study, there are several limitations: (1) The cross-sectional design focuses on associations rather than causal pathways. (2) The dataset lacked genetic and some perinatal information, which could have provided more profound insights into the predictors of depression and anxiety in Children with JIA. (3) The study did not account for different JIA subtypes, which could have varying impacts on mental health outcomes [10]. (4) Potential under-diagnosis of JIA in the comparison group might have led to underestimating the findings. (5) The results are based on children in the U.S., so caution is needed when generalizing these findings to other populations. Factors such as healthcare access [56], treatment, genetic predispositions [57], and sociocultural factors [58] may influence outcomes in ways that differ from those observed in U.S. children. Therefore, caution is warranted when applying these results to populations outside the U.S.

## CONCLUSION

This study underscores the vital interplay between JIA and mental health outcomes in children and adolescents. Specifically, we identified sex and insurance type as key modifiers influencing the risk of depression and anxiety, respectively. Among boys, JIA was associated with a threefold higher likelihood of depression, emphasizing the need to screen all children, especially those whose demographic profile might otherwise be considered “lower risk,” for mental health challenges. Meanwhile, children with JIA on public insurance were significantly more prone to anxiety than those on private insurance, revealing how socioeconomic factors and healthcare access can compound disease burden [18].

Building on these findings, it is paramount for healthcare providers, policymakers, and community organizations to develop multidimensional and equitable interventions. For instance, CBT shows promise for mitigating anxiety and depressive symptoms, especially when integrated early during JIA management [54]. Other known counseling interventions that assist with emotional regulation, anxiety, and depression include dialectical behavioral therapy (DBT) and acceptance and commitment therapy (ACT) [59-61]. These types of counseling interventions are effective because children and adolescents may need assistance to manage pain, emotional distress, and daily challenges. CBT teaches pain coping strategies, cognitive restructuring, and behavioral activation, while ACT promotes psychological flexibility through mindfulness and values-based living. DBT enhances distress tolerance, emotion regulation, and interpersonal skills, improving overall well-being despite chronic pain.

In addition to these therapeutic approaches, technological advancements in telemedicine and digital health applications can enhance access to mental health resources and provide continuous support for children and families managing juvenile arthritis. This benefit is particularly relevant for families relying on public insurance, as these services can help reduce barriers to care, such as transportation challenges, long wait times, and limited availability of specialists in underserved areas [51]. Telehealth platforms offer cost-effective alternatives by enabling virtual therapy sessions, remote pain management consultations, and digital mental health interventions, ensuring that children receive consistent and timely support without the financial strain of frequent in-person visits. The MBSR and family-based psychoeducation programs may further foster emotional regulation and resilient coping strategies [52, 53]. Addressing barriers linked to lower socioeconomic status, such as limited specialist availability or logistical hurdles, can help reduce disparities in mental healthcare usage [55]. Future investigations should employ longitudinal designs to clarify how JIA severity and treatment approaches influence mental health outcomes over time. A detailed examination of JIA subtypes, particularly those with higher susceptibility to psychiatric comorbidities, will deepen our understanding of these variations. Evaluating both genetic and environmental factors is also critical, as they may collectively moderate the relationship between JIA and mental well-being. Finally, to gauge the true impact of emerging interventions, such as digital cognitive behavioral therapy or remote monitoring, researchers must conduct robust cost-effectiveness analyses in larger, more diverse populations. By integrating medical, psychological, and socioeconomic considerations into routine care, we can optimize well-being and long-term disease outcomes for children and adolescents with JIA. Such a holistic approach promises not only to alleviate current burdens on young patients and their families but also to pave the way for healthier transitions into adulthood.

## Figures and Tables

**Table 1:** Demographic and health status of children with and without JIA.

	Juvenile Idiopathic Arthritis			
	No	Yes	Total	FDR^*p-value*
Total	191469	550	192019	
**Survey year**				0.1084
2016	42774 (22.3)	134 (24.4)	42908 (22.4)	
2017	18399 (9.6)	58 (10.6)	18457 (9.6)	
2018	26112 (13.6)	85 (15.5)	26197 (13.6)	
2019	25615 (13.4)	81 (14.7)	25696 (13.4)	
2020	36884 (19.3)	96 (17.3)	36979 (19.3)	
2021	41685 (21.8)	97 (17.6)	41782 (21.8)	
**Child’s age [Mean(SD)]**	10.43 (4.49)	13.40 (3.66)	11.00 (4.49)	<.0001*
**Sex**				<.0001*
Boy	99149 (51.8)	213 (38.7)	99362 (51.8)	
Girl	92320 (48.2)	337 (61.3)	92657 (48.2)	
**Race**				<.0001*
Hispanic	23579 (12.4)	55 (10.0)	23634 (12.3)	
White, non-Hispanic	130374 (68.3)	393 (71.6)	130767 (68.3)	
Black, non-Hispanic	12416 (6.5)	52 (9.5)	12468 (6.5)	
Asian, non-Hispanic	10241 (5.4)	8 (1.5)	10249 (5.4)	
American Indian or Alaska Native Non-Hispanic	1179 (0.6)	6 (1.1)	1185 (0.6)	
Others	13236 (6.9)	35 (6.4)	13271 (6.9)	
**Maternal age at delivery [Mean(SD)]**	30.10 (5.86)	29.11 (6.31)	30.00 (5.86)	0.0004*
**Premature birth**				
Yes	20593 (10.9)	96 (17.7)	20689 (10.9)	<.0001*
**Low birth weight**				<.0001*
No	166875 (91.5)	441 (85.1)	167316 (91.5)	
Low birth weight	13136 (7.2)	56 (10.8)	13192 (7.2)	
Very low birth weight	2429 (1.3)	21 (4.1)	2450 (1.3)	
**Months of breastfeeding**				<.0001*
6 months or longer, or still breastfeeding	18591 (9.7)	13 (2.4)	18604 (9.7)	
Children age 6-17 years	152969 (80.3)	520 (94.7)	153489 (80.3)	
Less than 6 months	19064 (10.0)	16 (2.9)	19080 (10.0)	
**BMI**				<.0001*
Normal weight	69167 (37.0)	231 (44.0)	69398 (37.0)	
Children age 4-9 years	81303 (43.5)	85 (16.2)	81388 (43.4)	
Underweight	6668 (3.6)	31 (5.9)	6699 (3.6)	
Overweight or obese	29831 (16.0)	178 (33.9)	30009 (16.0)	
**Sleeps less than recommended age-appropriate hours**				
Yes	57622 (30.5)	212 (39.8)	57834 (30.6)	<.0001*
**Depression**				
Yes	8643 (4.5)	117 (21.5)	8760 (4.6)	<.0001*
**Anxiety**				
Yes	19878 (10.5)	200 (36.6)	20078 (10.5)	<.0001*
**Allergic to food, drug, or insect**				
Yes	45198 (23.7)	266 (48.8)	45464 (23.7)	<.0001*
**Asthma**				
Yes	15925 (8.4)	140 (25.7)	16065 (8.4)	<.0001*
**Brain injury**				
Yes	684 (0.6)	13 (3.6)	697 (0.6)	<.0001*
**Behavior or conduct problem**				
Yes	14353 (7.5)	99 (18.1)	14452 (7.6)	<.0001*
**Developmental delay**				
Yes	10423 (5.5)	91 (16.8)	10514 (5.5)	<.0001*
**Intellectual disability**				
Yes	2107 (1.1)	33 (6.1)	2140 (1.1)	<.0001*
**Speech disability**				
Yes	10376 (5.4)	54 (9.9)	10430 (5.5)	<.0001*
**Learning disability**				
Yes	13821 (7.3)	125 (22.9)	13946 (7.3)	<.0001*
**Physical activity**				<.0001*
Everyday	32008 (16.9)	74 (13.8)	32082 (16.9)	
Children age 0-5 years	38500 (20.3)	30 (5.6)	38530 (20.3)	
4-6 days	45694 (24.1)	133 (24.8)	45827 (24.1)	
1-3 days	59025 (31.2)	200 (37.2)	59225 (31.2)	
0 day	14131 (7.5)	100 (18.6)	14231 (7.5)	
**Difficulties making or keeping friends**				<.0001*
0-5 years	38500 (20.3)	30 (5.6)	38530 (20.3)	
No difficulty	113811 (60.1)	268 (50.2)	114079 (60.1)	
A little difficulty	29418 (15.5)	153 (28.7)	29571 (15.6)	
A lot of difficulty	7607 (4.0)	83 (15.5)	7690 (4.1)	
**Insurance**				<.0001*
Uninsured	8406 (4.5)	26 (4.8)	8432 (4.5)	
Public	38326 (20.3)	180 (33.5)	38506 (20.4)	
Private	134464 (71.3)	286 (53.3)	134750 (71.3)	
Public and Private	7306 (3.9)	45 (8.4)	7351 (3.9)	
**Mother’s physical health status**				<.0001*
Excellent or very good	121311 (72.4)	212 (45.9)	121523 (72.4)	
Good	39160 (22.5)	170 (36.8)	39330 (23.1)	
Fair or poor	9331 (5.1)	80 (17.3)	9411 (5.5)	
**Father’s physical health status**				<.0001*
Excellent or very good	107151 (72.0)	197 (54.1)	107348 (71.9)	
Good	34738 (23.3)	125 (34.3)	34863 (23.4)	
Fair or poor	7030 (4.7)	42 (11.5)	7072 (4.7)	
**Mother’s mental health status**				<.0001*
Excellent or very good	125351 (73.8)	257 (55.6)	125608 (73.7)	
Good	34818 (20.5)	142 (30.7)	34960 (20.5)	
Fair or poor	9635 (5.7)	63 (13.6)	9698 (5.7)	
**Father’s mental health status**				<.0001*
Excellent or very good	117438 (78.9)	241 (66.2)	117679 (78.9)	
Good	25586 (17.2)	92 (25.3)	25678 (17.2)	
Fair or poor	5840 (3.9)	31 (8.5)	5871 (3.9)	
**Parents divorced or separated**				
Yes	45489 (24.5)	209 (39.6)	45698 (24.6)	<.0001*
**Parent died**				
Yes	6020 (3.3)	39 (7.4)	6059 (3.3)	<.0001*
**Parent served time in jail or prison**				
Yes	12239 (6.6)	68 (13.0)	12307 (6.6)	<.0001*
**Witnessed domestic violence**				
Yes	9880 (5.3)	69 (13.1)	9949 (5.4)	<.0001*
**Victim or witness of neighborhood violence**				
Yes	7143 (3.9)	67 (12.8)	7210 (3.9)	<.0001*
**Lived with anyone who was mentally ill, suicidal, or severely depressed**				
Yes	17840 (9.7)	115 (22.1)	17955 (9.7)	<.0001*
**Lived with anyone who had a problem with alcohol or drug**				
Yes	18846 (10.2)	109 (20.8)	18955 (10.2)	<.0001*
**Treated or judged unfairly because of their race or ethnic group**				
Yes	7229 (3.9)	43 (8.2)	7272 (3.9)	<.0001*

^FDR = false discovery rate

* p<0.05

**Table 2:** Multiple logistic regression for depression with and without JIA and Sex interaction.

	Model 1Depression		Model 2Depression with Effect Modifier	
	Odds Ratio (95% Confidence Interval)	*p-value*	Odds Ratio (95% Confidence Interval)	*p-value*
**Juvenile idiopathic arthritis (JIA)**				
No	1		--	--
Yes	1.22 (0.75 - 1.98)	0.4310	--	--
**Sex**				
Boys *vs*. Girls	0.52 (0.47 - 0.57)	<.0001*	--	--
**JIA ** × ** Sex**				
Among boys (JIA *vs*. Non-JIA)	--	--	2.96 (1.43 - 6.13)	0.0035*
Among girls (JIA *vs*. Non-JIA)	--	--	0.68 (0.35 - 1.32)	0.2529
Among Non-JIA (Boy *vs*. Girl)	--	--	0.51 (0.47 - 0.57)	<.0001*
Among JIA (Boy *vs*. Girl)	--	--	2.24 (0.84 - 6.01)	0.1079
**Child’s age [Mean(SD)]**	1.30 (1.27 - 1.34)	<.0001*	1.30 (1.27 - 1.34)	<.0001*
**Race**				
Asian, non-Hispanic	1		1	
Hispanic	1.77 (1.25 - 2.52)	0.0014*	1.78 (1.25 - 2.53)	0.0013*
White, non-Hispanic	2.10 (1.52 - 2.89)	<.0001*	2.11 (1.53 - 2.90)	<.0001*
Black, non-Hispanic	1.35 (0.88 - 2.07)	0.1658	1.35 (0.88 - 2.07)	0.1636
American Indian or Alaska Native Non-Hispanic	2.89 (1.56 - 5.37)	0.0008*	2.86 (1.54 - 5.32)	0.0009*
Others	1.99 (1.38 - 2.86)	0.0002*	2.00 (1.38 - 2.88)	0.0002*
**Maternal age at delivery [Mean(SD)]**	0.99 (0.98 - 1.00)	0.0286*	0.99 (0.98 - 1.00)	0.0259*
**Premature birth**				
Yes *vs*. No	1.16 (1.02 - 1.33)	0.0272*	1.16 (1.01 - 1.33)	0.0301*
**Months of breastfeeding**				
Less than 6 months	1		1	
Children age 6-17 years	3.04 (1.23 – 7.53)	0.0165*	2.99 (1.21 - 7.41)	0.0181*
6 months or longer, or still breastfeeding	0.18 (0.02 - 1.44)	0.1059	0.18 (0.02 - 1.44)	0.1059
**BMI**				
Children age 0-9 years	1		1	
Underweight	0.83 (0.61 - 1.13)	0.2280	0.83 (0.61 - 1.13)	0.2336
Normal weight	1.05 (0.83 - 1.34)	0.6745	1.05 (0.83 - 1.34)	0.6662
Overweight or obese	1.30 (1.02 - 1.65)	0.0333*	1.30 (1.02 - 1.66)	0.0316*
**Sleeping less than recommended age-appropriate hours**				
Yes *vs*. No	1.26 (1.14 - 1.38)	<.0001*	1.26 (1.14 - 1.39)	<.0001*
**Allergic to food, drug, or insect**				
Yes *vs*. No	1.36 (1.23 - 1.51)	<.0001*	1.36 (1.23 - 1.51)	<.0001*
**Asthma**				
Yes *vs*. No	1.52 (1.33 - 1.74)	<.0001*	1.52 (1.33 - 1.74)	<.0001*
**Brain injury**				
Yes *vs*. No	1.26 (0.86 - 1.85)	0.2356	1.28 (0.87 - 1.88)	0.2090
**Behavior or conduct problem**				
Yes *vs*. No	4.20 (3.69 - 4.78)	<.0001*	4.20 (3.69 - 4.79)	<.0001*
**Developmental delay**				
Yes *vs*. No	1.30 (1.06 - 1.59)	0.0128*	1.30 (1.06 - 1.60)	0.0116*
**Intellectual disability**				
Yes *vs*. No	3.66 (2.55 - 5.25)	<.0001*	3.69 (2.57 - 5.30)	<.0001*
**Speech disability**				
Yes *vs*. No	1.69 (1.33 - 2.13)	<.0001*	1.67 (1.32 - 2.11)	<.0001*
**Learning disability**				
Yes *vs*. No	1.54 (1.32 - 1.79)	<.0001*	1.54 (1.32 - 1.79)	<.0001*
**Physical activity**				
0 day	1		1	
Children age 0-5 years	0.38 (0.24 - 0.63)	0.0001*	0.38 (0.23 - 0.62)	0.0001*
1-3 days	2.10 (1.76 - 2.51)	<.0001*	2.10 (1.75 - 2.51)	<.0001*
Everyday	1.44 (1.23 - 1.69)	<.0001*	1.44 (1.23 - 1.68)	<.0001*
**Difficulties making or keeping friends**				
A lot of difficulty	1		1	
0-5 years	0.86 (0.62 - 1.18)	0.3397	0.85 (0.62 - 1.17)	0.3085
No difficulty	0.12 (0.10 - 0.13)	<.0001*	0.12 (0.10 - 0.13)	<.0001*
**Insurance type**				
Uninsured	1		1	
Public	1.67 (1.25 - 2.23)	0.0006*	0.85 (0.62 - 1.17)	0.3085
Private	1.36 (1.03 - 1.79)	0.0277*	0.12 (0.10 - 0.13)	<.0001*
Public and Private	2.02 (1.45 - 2.81)	<.0001*	2.02 (1.45 - 2.81)	<.0001*
**Father’s physical health status**				
Fair or poor	1		1	
Good	1.40 (1.15 - 1.70)	0.0007*	1.41 (1.16 - 1.72)	0.0005*
Excellent or very good	1.17 (0.97 - 1.42)	0.0972	1.18 (0.98 - 1.43)	0.0811
**Mother’s mental health status**				
Fair or poor	1		1	
Good	0.44 (0.38 - 0.52)	<.0001*	0.44 (0.38 - 0.52)	<.0001*
Excellent or very good	0.70 (0.60 - 0.83)	<.0001*	0.70 (0.60 - 0.83)	<.0001*
**Father’s mental health status**				
Fair or poor	1		1	
Good	0.60 (0.49 - 0.73)	<.0001*	0.60 (0.49 - 0.73)	<.0001*
Excellent or very good	0.86 (0.71 - 1.04)	0.1173	0.86 (0.71 - 1.04)	0.1277
**Parents divorced or separated**				
Yes *vs*. No	1.52 (1.27 - 1.81)	<.0001*	1.51 (1.26 - 1.80)	<.0001*
**Victim or witness of neighborhood violence**				
Yes *vs*. No	1.32 (1.18 - 1.48)	<.0001*	1.33 (1.18 - 1.49)	<.0001*
**Lived with anyone who was mentally ill, suicidal, or severely depressed**				
Yes *vs*. No	1.95 (1.72 - 2.21)	<.0001*	1.96 (1.73 - 2.22)	<.0001*
**Lived with anyone who had a problem with alcohol or drug**				
Yes *vs*. No	1.15 (0.99 - 1.31)	0.0549	1.14 (0.99 - 1.31)	0.0607

* p<0.05

**Table 3:** Multiple logistic regression for anxiety with and without JIA and insurance interaction.

	Model 1Anxiety		Model 2Anxiety with Effect Modifier	
	Odds Ratio (95% Confidence Interval)	*p-value*	Odds Ratio (95% Confidence Interval)	*p-value*
**Juvenile idiopathic arthritis (JIA)**				
No	1		--	--
Yes	1.66 (1.13 - 2.44)	0.0092*	--	--
**Insurance type**				
Uninsured	1		--	--
Public	1.72 (1.39 - 2.12)	<.0001*	--	--
Private	1.77 (1.46 - 2.16)	<.0001*	--	--
Public and Private	2.15 (1.69 - 2.73)	<.0001*	--	--
**JIA ** × ** Insurance type**				
Among uninsured (JIA *vs*. Non-JIA)	--	--	0.60 (0.03 - 13.18)	0.7474
Among public insurance (JIA *vs*. Non-JIA)	--	--	6.28 (2.83 - 13.90)	<.0001*
Among private insurance (JIA *vs*. Non-JIA)	--	--	1.14 (0.70 - 1.86)	0.5883
Among public and private insurance (JIA *vs*. Non-JIA)	--	--	0.79 (0.16 - 3.85)	0.7738
Among Non-JIA (Public *vs*. Uninsured)	--	--	1.69 (1.37 - 2.08)	<.0001*
Among Non-JIA (Private *vs*. Uninsured)	--	--	1.77 (1.45 - 2.15)	<.0001*
Among Non-JIA (Public and Private *vs*. Uninsured)	--	--	2.15 (1.69 - 2.73)	<.0001*
Among Non-JIA (Public *vs*. Private)	--	--	0.95 (0.87 - 1.05)	0.3228
Among Non-JIA (Public *vs*. Public and Private)	--	--	0.79 (0.67 - 0.92)	0.0034*
Among Non-JIA (Private *vs*. Public and Private)	--	--	0.82 (0.71 - 0.95)	0.0093*
Among JIA (Public *vs*. Uninsured)	--	--	17.58 (0.73 - 422.09)	0.0772
Among JIA (Private *vs*. Uninsured)	--	--	3.36 (0.15 - 75.92)	0.4463
Among JIA (Public and Private *vs*. Uninsured)	--	--	2.83 (0.09 - 89.83)	0.5554
Among JIA (Public *vs*. Private)	--	--	5.23 (2.07 - 13.25)	0.0005*
Among JIA (Public *vs*. Public and Private)	--	--	6.21 (1.07 - 36.19)	0.0423*
Among JIA (Private *vs*. Public and Private)	--	--	1.19 (0.23 - 6.17)	0.8387
**Child’s age [Mean(SD)]**	1.10 (1.09 - 1.11)	<.0001*	1.10 (1.09 - 1.11)	<.0001*
**Sex**				
Boys *vs*. Girls	0.58 (0.55 - 0.62)	<.0001*	0.58 (0.55 - 0.62)	<.0001*
**Race**				
Asian, non-Hispanic	1		1	
Hispanic	2.59 (2.04 - 3.29)	<.0001*	2.58 (2.03 - 3.28)	<.0001*
White, non-Hispanic	3.61 (2.89 - 4.50)	<.0001*	3.60 (2.89 - 4.49)	<.0001*
Black, non-Hispanic	1.52 (1.13 - 2.04)	0.0052*	1.52 (1.13 - 2.04)	0.0053*
American Indian or Alaska Native Non-Hispanic	3.49 (2.21 - 5.51)	<.0001*	3.45 (2.19 - 5.45)	<.0001*
Others	2.64 (2.06 - 3.39)	<.0001*	2.64 (2.06 - 3.39)	<.0001*
**Premature birth**				
Yes *vs*. No	1.21 (1.10 - 1.34)	0.0002*	1.21 (1.10 - 1.34)	0.0002*
**Low birth weight**				
No	1		1	
Low birth weight	0.90 (0.79 - 1.03)	0.1234	0.91 (0.80 - 1.03)	0.1270
Very low birth weight	0.77 (0.59 - 1.01)	0.0602	0.78 (0.59 - 1.02)	0.0677
**Months of breastfeeding**				
Less than 6 months	1		1	
Children age 6-17 years	2.71 (1.87 - 3.92)	<.0001*	2.70 (1.87 - 3.90)	<.0001*
6 months or longer, or still breastfeeding	0.86 (0.66 - 1.14)	0.2960	0.86 (0.66 - 1.14)	0.2992
**Sleeping less than recommended age-appropriate hours**				
Yes *vs*. No	1.11 (1.04 - 1.18)	0.0020*	1.11 (1.04 - 1.18)	0.0020*
**Allergic to food, drug, or insect**				
Yes *vs*. No	1.69 (1.59 - 1.81)	<.0001*	1.70 (1.59 - 1.81)	<.0001*
**Asthma**				
Yes *vs*. No	1.36 (1.24 - 1.49)	<.0001*	1.36 (1.24 - 1.49)	<.0001*
**Brain injury**				
Yes *vs*. No	1.55 (1.18 - 2.04)	0.0017*	1.56 (1.19 - 2.05)	0.0015*
**Behavior or conduct problem**				
Yes *vs*. No	4.11 (3.75 - 4.49)	<.0001*	4.11 (3.75 - 4.49)	<.0001*
**Developmental delay**				
Yes *vs*. No	0.78 (0.69 - 0.89)	0.0003*	0.79 (0.69 - 0.90)	0.0003*
**Intellectual disability**				
Yes *vs*. No	2.15 (1.73 - 2.69)	<.0001*	2.16 (1.73 - 2.69)	<.0001*
**Learning disability**				
Yes *vs*. No	1.88 (1.70 - 2.09)	<.0001*	1.89 (1.70 - 2.10)	<.0001*
**Physical activity**				
0 day	1		1	
Children age 0-5 years	0.45 (0.34 - 0.61)	<.0001*	0.45 (0.34 - 0.60)	<.0001*
1-3 days	1.79 (1.59 - 2.01)	<.0001*	1.79 (1.59 - 2.01)	<.0001*
Everyday	1.30 (1.19 - 1.42)	<.0001*	1.30 (1.19 - 1.43)	<.0001*
**Difficulties making or keeping friends**				
A lot of difficulty	1		1	
0-5 years	0.66 (0.52 - 0.84)	0.0008*	0.66 (0.52 - 0.84)	0.0007*
No difficulty	0.16 (0.14 - 0.18)	<.0001*	0.16 (0.14 - 0.18)	<.0001*
**Father’s physical health status**				
Fair or poor	1		1	
Good	1.24 (1.08 - 1.42)	0.0024*	1.24 (1.08 - 1.42)	0.0022*
Excellent or very good	0.99 (0.87 - 1.13)	0.8916	0.99 (0.87 - 1.14)	0.9206
**Mother’s mental health status**				
Fair or poor	1		1	
Good	0.60 (0.53 - 0.68)	<.0001*	0.60 (0.53 - 0.68)	<.0001*
Excellent or very good	0.93 (0.82 - 1.06)	0.2704	0.93 (0.82 - 1.06)	0.2677
**Father’s mental health status**				
Fair or poor	1		1	
Good	0.67 (0.58 - 0.78)	<.0001*	0.67 (0.58 - 0.78)	<.0001*
Excellent or very good	0.87 (0.75 - 1.00)	0.0544	0.87 (0.75 - 1.01)	0.0577
**Parent served time in jail or prison**				
Yes *vs*. No	0.83 (0.71 - 0.98)	0.0244*	0.83 (0.71 - 0.97)	0.0218*
**Witnessed domestic violence**				
Yes *vs*. No	0.85 (0.73 - 0.99)	0.0346*	0.85 (0.73 - 0.99)	0.0381*
**Victim or witness of neighborhood violence**				
Yes *vs*. No	1.45 (1.25 - 1.68)	<.0001*	1.44 (1.25 - 1.68)	<.0001*
**Lived with anyone who was mentally ill, suicidal, or severely depressed**				
Yes *vs*. No	1.76 (1.61 - 1.93)	<.0001*	1.76 (1.61 - 1.93)	<.0001*
**Lived with anyone who had a problem with alcohol or drug**				
Yes *vs*. No	1.11 (1.00 - 1.24)	0.0557	1.11 (1.00 - 1.24)	0.0559
**Treated or judged unfairly because of their race or ethnic group**				
Yes *vs*. No	1.23 (1.05 - 1.44)	0.0105*	1.23 (1.05 - 1.44)	0.0094*

* p<0.05

## Data Availability

The anonymized NSCH data collected are available as open databases *via* https://www.childhealthdata.org/dataset/download?rq=16239.

## LIST OF ABBREVIATIONS

ABE: = Augmented backward elimination
FDR: = False discovery rate
JIA: = Juvenile idiopathic arthritis
NSCH: = National Survey of Children’s Health
NS-CSHCN: = National Survey of Children with Special Health Care Needs
SES: = Socioeconomic status
SES: = Socioeconomic status
BMI: = Body mass index
FDR: = False discovery rate
ABE: = Augmented Backward Elimination
CBT: = Cognitive Behavioral Therapy
MBSR: = Mindfulness-Based Stress Reduction

## STANDARDS OF REPORTING

STROBE guidelines were followed.
